# Editorial Comment: Robotic versus other nephroureterectomy techniques: a systematic review and meta-analysis of over 87,000 cases

**DOI:** 10.1590/S1677-5538.IBJU.2020.02.11

**Published:** 2020-01-10

**Authors:** João Paulo Martins de Carvalho

**Affiliations:** 1 Serviço de Urologia Hospital Federal Cardoso Fontes Rio de JaneiroRJ Brasil Serviço de Urologia, Hospital Federal Cardoso Fontes, Rio de Janeiro, RJ, Brasil

Veccia A ^1,2^, Antonelli A ^2^, Francavilla S ^2^, Simeone C ^2^, Guruli G ^1^, Zargar H ^3^, Perdoná S ^4^, Ferro M ^5^, Carrieri G ^6^, Hampton LJ ^1^, Porpiglia F ^7^, Autorino R ^8^

*^1^*Division of Urology, VCU Health System, VCU Medical Center, PO Box 980118, Richmond, VA, 23298-0118, USA; ^2^ Urology Unit, ASST Spedali Civili Hospital, Department of Medical and Surgical Specialties, Radiological Science, and Public Health, University of Brescia, Brescia, Italy; ^3^ Department of Surgery, Department of Urology, University of Melbourne, Royal Melbourne Hospital, Melbourne, Australia; ^4^ Uro-Gynecological Department, Fondazione “G. Pascale” IRCCS, Naples, Italy; ^5^ Division of Urology, European Institute of Oncology, Milan, Italy; ^6^ Urology and Renal Transplantation Unit, Department of Medical and Surgical Sciences, University of Foggia, Foggia, Italy; ^7^ Division of Urology, San Luigi Gonzaga Hospital, Orbassano, Turin, Italy; ^8^ Division of Urology, VCU Health System, VCU Medical Center, PO Box 980118, Richmond, VA, 23298-0118, USA

World J Urol. 2019 Nov 26 [Epub ahead of print]

**DOI:** 10.1007/s00345-019-03020-1 | **ACCESS:** 10.1007/s00345-019-03020-1

## COMMENT

This is a meta-analysis of the latest publications on robotic nephro ureterectomy (RNU) comparing it with other techniques. Through research of publications over the last 20 years until April 2019. The authors were able to filter 80 studies as eligible for research. They performed a historical review that adopted nephro ureterectomy with bladder cuff removal as the gold standard for curable resectability in high-grade upper urinary tract urothelial carcinoma.

Over the years, open techniques have been replaced by laparoscopic and laparoscopic robotic assisted. However, robust data are lacking comparing the different techniques regarding intra and postoperative results. Thus, these variables were evaluated in the most different studies: age, gender, body mass index (BMI), race (Caucasian), Charlson Comorbidity Index (CCI)≥2, American Society of Anesthesiologist (ASA) score≥3, tumor location (pelvicalyceal, ureteral, multifocal), and surgery performed in an academic hospital; – surgical outcomes: estimated blood loss (EBL), operative time (OT), intraoperative complications, transfusions, overall complications, major complications (Clavien≥3), and length of stay; – pathological outcomes: pT≥3, high-grade tumor, pN+, nodes removed, and positive surgical margins (PSM); – survival outcomes: recurrence, metastasis, death, 2- and 5-years recurrence free survival (RFS), 2- and 5-years cancer specifc survival (CSS), and correlation between surgical technique and RFS and CSS.

The authors than compared the open nephroureterectomy (ONU) , pure laparoscopic (LNU), laparoscopic hand assisted ( HALNU), laparoscopic assisted robot (RNU).

The authors mention that over the last 19 years (2000-2019) there has been an increase of up to 36% with series for the procedure performed by (RNU) including the 80 selected articles.

There was no difference between the initial criteria adopted in the different studies. Bladder cuff extraction was performed intracorporeally in all series with RNU, and in 50% of the LNU. Lower bleeding rate in the RNU and higher in the open. Less surgical time at the ONU and no difference between the other techniques. RNU with a lower rate of intraoperative complications and no difference in blood transfusion between RNU and LNU. RNU was more frequently performed in patients with high-risk cancer, but without pathological differences between specimens, but HALNU documented the lowest rate of positive margins as well as the lowest recurrence rate and metastasis. The ONU had the lowest cancer-specific survival rate in 05 years. Intracorporeal Bladder CUFF extraction documented the lowest local recurrence rate.

Another series report with 78 patients who underwent RNU in high volume centers, from 2008 to 2017, on the Si and Xi platforms. It presented similar results in cancer control and progression-free survival. However, as documented in this study, no comparisons were made between the different techniques (1).

The authors cite that this was the largest published meta-analysis of 87,000 patients, with no differences between studies in their baseline criteria or tumor location. Robotic bladder cuff extraction has lower transfusion rates when compared to other techniques. No difference in transfusions between RNU and LNU, no time difference in RNU when compared to other techniques. Much of the decrease in surgical time in the RNU has been attributed to improved platforms. Long-term complications were equivalent for all techniques and shorter hospital stay.

However, this data may be biased, considering the routine of different services at the time of hospitalization. The RNU also showed a higher number of dissected lymph nodes compared to other techniques. The different techniques did not present statistically significant differences for local recurrences, with a lower intra-bladder recurrence rate for RNU. Although there was a tendency for a higher rate of metastasis to the RNU, however, these patients had a higher risk disease.

Finally, the authors argue that all techniques have similar long-term oncological outcomes with a larger long-term URI trend in view of the popularity of the technique. There is a need for specific cancer survival studies among the different techniques.
